# Comparative Proteomic Analysis of Two *Ralstonia solanacearum* Isolates Differing in Aggressiveness

**DOI:** 10.3390/ijms19082444

**Published:** 2018-08-18

**Authors:** Guoping Wang, Jie Kong, Dandan Cui, Hongbo Zhao, Puyan Zhao, Shujie Feng, Yahua Zhao, Wenyi Wang

**Affiliations:** 1 Key Laboratory of Biology and Germplasm Enhancement of Horticultural Crops in South China, Ministry of Agriculture, College of Horticulture, South China Agricultural University, Guangzhou 510642, China; gpwang@scau.edu.cn (G.W.); cuidan0627@163.com (D.C.); 2 College of Horticulture, South China Agricultural University, Guangzhou 510642, China; zhao@scau.edu.cn (H.Z); zhaopuyan@scau.edu.cn (P.Z.); sjief@scau.edu.cn (S.F.); 3 Key Laboratory of Protein Function and Regulation in Agricultural Organisms, College of Life Sciences, South China Agricultural University, Guangzhou 510642, China; kongjiechn@foxmail.com; 4 Department of Plant Science, Weizmann Institute of Science, Rehovot 76100, Israel

**Keywords:** *Ralstonia solanacearum*, UDP-N-acetylglucosamine 2-epimerase (epsC), isocitrate lyase (ICL), pathogenesis, proteome

## Abstract

*Ralstonia solanacearum* is a soil-borne, plant xylem-infecting pathogen that causes the devastating bacterial wilt (BW) disease in a number of plant species. In the present study, two *R. solanacearum* strains with different degrees of aggressiveness―namely RsH (pathogenic to Hawaii 7996, a tomato cultivar resistant against most strains) and RsM (non-pathogenic to Hawaii 7996) were identified. Phylogenetic analysis revealed that both RsM and RsH belonged to phylotype I. To further elucidate the underlying mechanism of the different pathotypes between the two strains, we performed a comparative proteomics study on RsM and RsH in rich and minimal media to identify the change in the level of protein abundance. In total, 24 differential proteins were identified, with four clusters in terms of protein abundance. Further bioinformatics exploration allowed us to classify these proteins into five functional groups. Notably, the pathogenesis of RsM and RsH was particularly characterized by a pronounced difference in the abundance of virulence- and metabolism-related proteins, such as UDP-N-acetylglucosamine 2-epimerase (epsC) and isocitrate lyase (ICL), which were more abundant in the high pathogenicity strain RsH. Thus, we propose that the differences in pathogenicity between RsM and RsH can possibly be partially explained by differences in extracellular polysaccharide (EPS) and glyoxylate metabolism-related proteins.

## 1. Introduction

*Ralstonia solanacearum* is the main causal agent of bacterial wilt (BW) disease, which is considered to be one of the most destructive plant diseases in tropical, subtropical, and warm-temperature regions [1]. Some cryophilic strains of this pathogen were isolated from Europe and North America during the early 1990s [2,3]. A recent survey was conducted by 458 bacterial pathologists worldwide to vote on the top 10 most pathogenic bacteria in terms of their scientific and economic importance and, of these, *R. solanacearum* claimed second place in the list [4]. *R. solanacearum* is a soil-borne pathogen that enters the plant roots through openings, such as wounds, and then it moves rapidly throughout the vascular system, leading to lethal generalized wilting [5,6]. It infects a range of plant species, including some important horticultural plants such as eggplants (*Solanum melongena*), potatoes (*Solanum tuberosum*), and tomatoes (*Solanum lycopersicum*).

Tomatoes are susceptible to infection by *R. solanacearum*, and infection can lead to substantial production losses, particularly in tropical and subtropical countries [6]. Moreover, *R. solanacearum* can colonize the weed rhizosphere, which does not cause symptoms, thereby allowing the plant to survive over seasons in the tomato fields [7]. Breeding resistant and tolerant tomato cultivars is still considered to be the most effective strategy for controlling this disease. So far, several key quantitative trait loci (QTLs) have been identified in tomatoes [7,8,9,10]. In keeping pace with the development of biological tools, proteomics are considered to be a powerful tool to determine pathogenesis-related proteins that may contribute to specific functions in pathogenic bacterium and fungi, such as *Aspergillus flavus*, *Ustilago maydis*, *Fusarium graminearum*, and *Botrytis cinerea* [11,12,13]. Hence, comprehensive proteomic analysis of different *R. solanacearum* strains exhibiting different levels of pathogenicity will allow us to unravel the mechanism of pathogenicity, to help in developing more durable and resistant tomato plants.

Owing to the importance of *R. solanacearum*, its pathogenicity has been extensively studied in the past decades. During *R. solanacearum*–plant interaction, several traits have been shown to contribute toward the virulence of *R. solanacearum* strains. Currently, regulation of pathogenicity in *R. solanacearum* focuses on a pathogenicity (Hrp) type III secretion system (T3SS), which is essential for pathogenicity [14]. T3SS is encoded by a set of *hrp* genes. Via this system, plant pathogenic bacteria deliver 15–30 effectors into the host cell to allow bacterial infection by exploiting the plant’s signaling pathways, thereby suppressing the plant’s defense systems [15,16]. Apart from T3SS, other factors such as extracellular polysaccharide (EPS), the type two secretion system (T2SS), lipopolysaccharide (LPS), and lectins also play crucial roles in the process of pathogenicity [17,18,19].

*R. solanacearum* is the most destructive bacterial plant pathogen, in part because it comprises a very broad range of strains varying in their geographical origins, host ranges, and pathogenicity determinants [4]. As a root and vascular pathogen, *R. solanacearum* is a model system to study bacterial pathogenicity [4]. However, as a plant pathogen that comprises a “species complex”, its regulatory network with respect to pathogenicity determinants in still unclear. In the present study, we identified two *R. solanacearum* stains: RsM (mildly aggressive strain) and RsH (a pathogenic, highly aggressive strain) to infect the tomato breeding line “Hawaii 7996”, which is the most widely used bacterial wilt-resistant cultivar [20]. Two-dimensional gel electrophoresis (2-DE) and electron spray ionization-mass spectrometry (ESI-MS/MS) were performed to identify differentially abundant proteins under various growth conditions, a total of 24 differential protein spots were identified with four clusters in terms of abundance level, of which 11 were matched proteins with annotated functions. Notably, the lethal pathotype RsH showed increased abundance of UDP-N-acetylglucosamine 2-epimerase (epsC) and isocitrate lyase (ICL), whereas the mild pathotype RsM harbored enhanced levels of membrane proteins. Because proteins are directly associated with biological functional systems, the results of this study will help to elucidate the pathogenicity or virulence factors of *R. solanacearum*.

## 2. Results

### 2.1. Evaluation of RsM and RsH Infection in the Hawaii 7996 Cultivar

**Figure 1:**
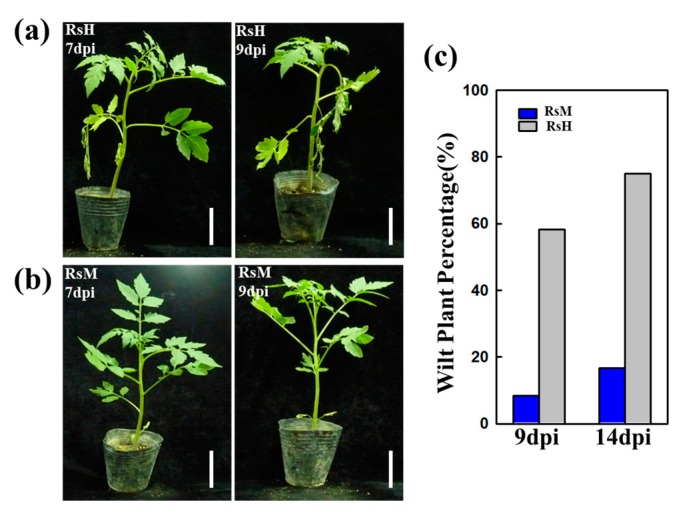
Susceptibilities of the tomato cultivar “Hawaii 7996” to *R. solanacearum* strains RsH (**a**,**c**) and RsM (**b**,**c**). Disease symptom evaluation after RsH and RsM infection in Hawaii 7996 at 9 dpi and 14 dpi (**c**). Scar bar = 10 cm.

“Hawaii 7996” (*Solanum lycopersicum*) is a well-known, stable tomato cultivar that is resistant to a range of *R. solanacearum* strains. Initially, two *R. solanacearum* strains, the highly aggressive isolate RsH and the weakly aggressive isolate RsM, were used to infect tomato crops with three independent biological replicates of each isolate. As reported in Figure 1, the tomato plants showed significantly different responses after RsM and RsH infection. At 7 dpi, typical wilt symptoms were observed after RsH infection (Figure 1a), whereas the tomato plants infected with RsM did not show any change until 9 dpi (Figure 1b), leading to almost full resistance to RsM (Figure 1b). Moreover, tomato leaves inoculated with RsH displayed highly susceptibility with typical external symptoms, namely leaf wilting after 7 dpi (Figure 1c). Our study revealed that RsH is an aggressive virulent variant of *R. solanacearum*, because Hawaii 7996 is stably resistant against most of the isolates in different environments.

### 2.2. Characteristics of Two R. solanacearum Strains: RsM and RsH

**Figure 2:**
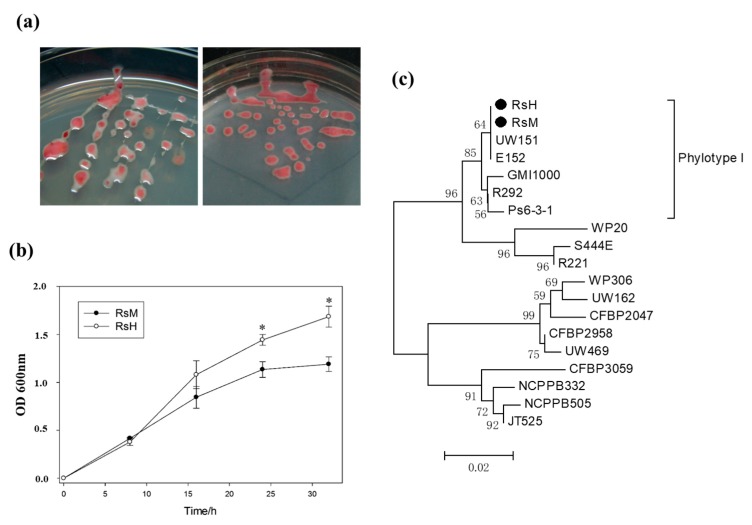
Morphological characterization and phylotype identification of RsM and RsH. (**a**) Phenotypes of the *R. solanacearum* strains RsM and RsH growing on TZC agar plates after 48 h; (**b**) production of RsM and RsH was examined by measuring the absorbance at 600 nm at different time point intervals. Values are expressed as means ± SD (* *p* < 0.05), and were compared with RsM using a Student’s *t*-test; (**c**) phylogenetic trees are generated according to the sequence homology of *egl* genes by MEGA5 software, using the neighbor-joining method. The bar represents a two nucleotide change per 100 nucleotide positions. A “●” is used as a proxy of “RsH” and “RsM” to indicate the stains used in this analysis. The *egl* sequences of S444E, R221, WP20, E152, R292, Ps6-3-1, GMI1000, UW151, UW469, CFBP2958, CFBP2047, UW162, WP306, JT525, NCPPB505, NCPPB332, and CFBP3059 were used [21].

To better characterize RsM and RsH, the morphological characteristics of RsM and RsH were monitored using Kelman’s tetrazolium chloride (TZC) medium after 48 h. RsM exhibited an obvious white outer edge compared with RsH. Moreover, both RsM and RsH displayed mucoid colonies with an irregular surface (Figure 2a) due to accumulation of exopolysaccharide (EPS).

Subsequently, the proliferation rate of both *R. solanacearum* isolates was measured after 8, 16, 24, and 32 h of incubation in a minimal liquid medium. The optical density of both the strains was calculated from three biological replications. In the first 8 h, the strains did not exhibit significant differences (Figure 2b). RsM proliferated gradually after 8 h of inoculation, resulting in a lower bacterial concentration compared with RsH (Figure 2b). Thus, RsM exhibited a significantly lower proliferation rate. We concluded that RsM and RsH differ both in morphological characteristics and proliferation rate. To determine the relationship between RsM and RsH, we also performed phylotype analysis for both strains.

### 2.3. Phylotype Identification of RsM and RsH

A classification system was developed by Fegan and Prior [22] based upon *hrpB* and *endoglucanase (egl)* genes. Using this method, we rapidly identified the phylotype of an *R. solanacearum* 759/760 primer pair. All strains exhibited a 280-bp complex-specific fragment, and phylotypes I, II, III, and IV yielded conserved phylotype-specific amplification bands of 144, 372, 91, and 213 bp, respectively (Figure S1). Furthermore, the *hrpB* and *egl* sequences in RsM and RsH were analyzed, and >99% sequence similarity was observed between these two strains (Figure S2), suggesting that both RsM and RsH are phylotype I and are closely related. As a consequence, we generated a phylogenetic tree based on the sequence homology of *egl* (Figure 2c). The resulting tree revealed that RsM and RsH are most closely related to strains UW151 (race1/biovar4) and E152 (race1/biovar3), which belong to phylotype I. Taken together, the results of the morphological characteristics and proliferation rate confirmed that RsM and RsH are closely related and exhibit different degrees of virulence in tomato plants.

### 2.4. Proteome Analysis of the Two R. solanacearum Strains RsM and RsH

**Figure 3:**
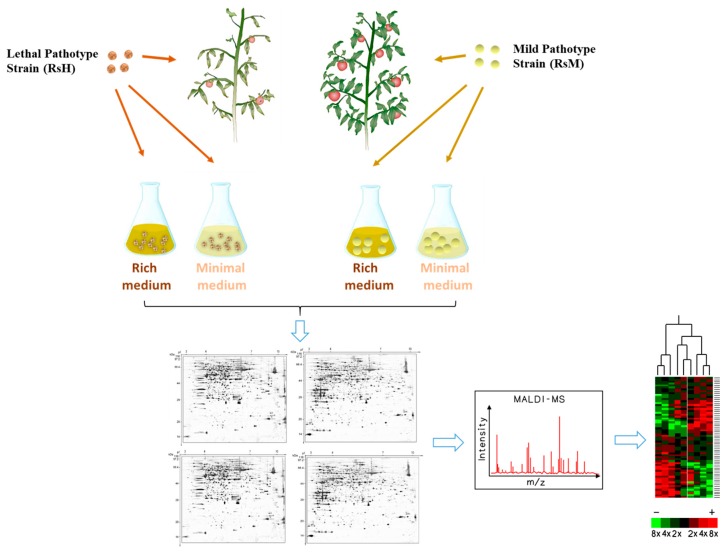
Experimental design. The highly aggressive isolate RsH and the weakly aggressive isolate RsM were inoculated in minimal (Min) and rich CPZ medium, then four kinds of proteins (Min-RsH, Min-RsM, CPZ-RsM, CPZ-RsH) were extracted from the bacterial harvest and used for two-dimensional gel electrophoretograms (2-DE) analysis.

**Figure 4:**
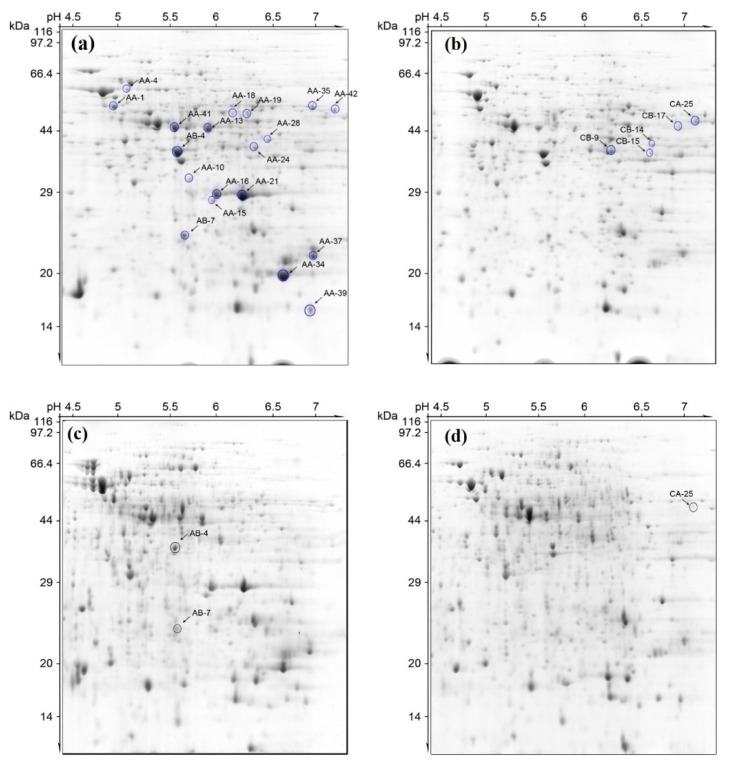
2-DE pattern of the proteins isolated from the *R. solanacearum* strains RsM and RsH in Min and CPZ media. (**a**) RsH in minimal medium; (**b**) RsM in minimal medium; (**c**) RsH in CPZ medium; (**d**) RsM in CPZ medium.

To better understand the regulation of the pathogenicity mechanism underlying *R. solanacearum* infection, we used two media—minimal (Min) and rich CPZ medium—to inoculate the two *R. solanacearum* strains of interest (Figure 3, Figure S3). CPZ medium was selected as a neutral baseline for comparison to the minimal medium, with limited nutritional value, to mimic the plant environment [21,22]. Hence, four kinds of proteins (Min-RsH, Min-RsM, CPZ-RsM, CPZ-RsH) were isolated from the bacterial harvest and were subjected to 2-DE analysis (Figure 4).

As a result, approximately 490 reproducible protein spots were detected in each Coomassie brilliant blue (CBB)-stained gel, in the range of pH 4–7 and with relative molecular masses of 14–97 kDa, using PDQuest software (Figure 4). In total, 24 proteins were identified with differential abundance in these samples, using matrix-assisted laser desorption/ionization time-of-flight (MALDI-TOF/MS-MS). All the identified proteins in different samples were classified into four clusters in terms of protein abundance (Figure S4). All secret protein spots are listed in Table 1, along with detailed information (Table 1, Table S3).

Cluster 1: Represents strain RsH being more abundant compared to RsM in both CPZ and Min media. A total of nine proteins were isolated from this group, including trigger factor (spot AA-1), Heat shock protein 60 family chaperone GroEL (spot AA-4), isocitrate dehydrogenase (IDH, spot AA-13), methyltransferase (spot AA-15), isocitrate lyase (ICL, spot AA-18), isocitrate dehydrogenase (IDH, spot AA-13), and more. Importantly, out of the isolated proteins, isocitrate dehydrogenase (IDH) and ICL shared a common substrate which was involved in the tricarboxylic acid (TCA) and glyoxylate cycles, indicating that the TCA and glyoxylate cycles participated in bacterial pathogen virulence. This result was consistent with a previous report stating that ICL is essential for successful colonization.

Cluster 2: Represents differential abundance of proteins only in RsH-Min, compared with that of proteins in RsM-Min, RsM-CPZ, and RsH-CPZ, including UDP-N-acetylglucosamine 2-epimerase (epsC, spot AA-24), hemin transport protein (spot AA-28), polyphenol oxidase B (spot AA-42), and more. Notably, epsC, a component of EPS I, is considered to be the major factor accounting for the virulence of the pathogen.

Cluster 3: Represents the abundant proteins in RsM-Min and RsH-Min, compared with those in RsM-CPZ and RsH-CPZ. Surprisingly, only three proteins were isolated for this group, including isocitrate dehydrogenase (IDH, spot AA-41), ornithine cyclodeamin (OCD, spot AB-4), and hypothetical protein RSc0416 (spot AB-7).

Cluster 4: Represents strain RsM exhibiting more abundance compared with RsH in both CPZ and Min medium. This group included five proteins, namely UDP-N-acetylglucosamine 2-epimerase (epsC, spot CB-9), glutamate carboxypeptidase (GPC, spot CB-14), diaminopimelate decarboxylase (DAPDC, spot CB-17) and two porin proteins (spots CB-15 and CA-25). We noticed that most of the proteins belonging to this group were membrane proteins such as GPC and porin, suggesting that membrane proteins may contribute to low bacterial virulence during tomato infection.

### 2.5. Protein Identification and Gene Ontology (GO) Annotation

**Figure 5:**
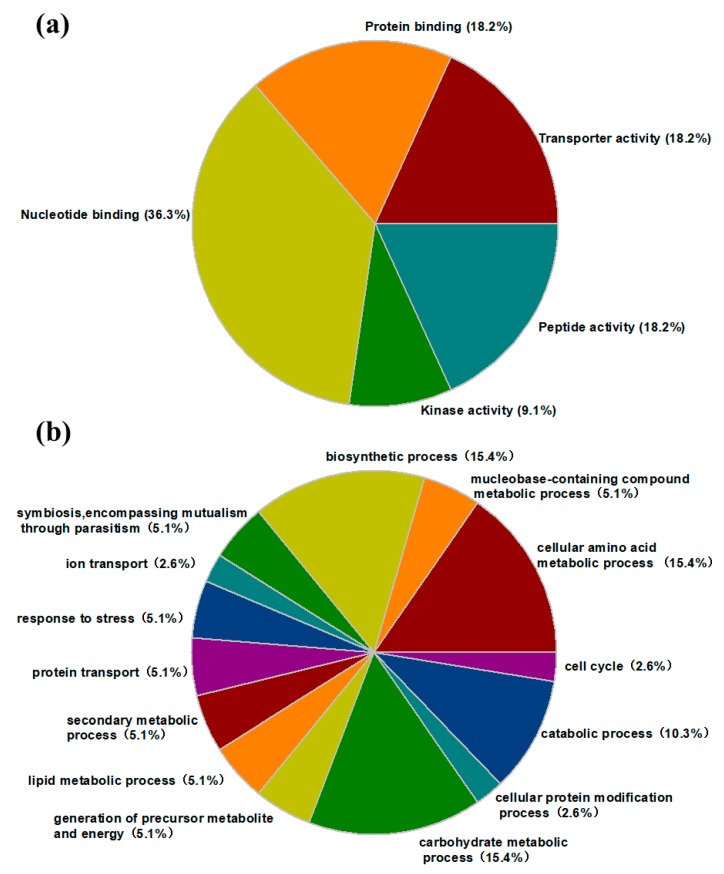
Functional classification of the differentially abundant proteins in terms of protein molecular functions (**a**) and biological processes (**b**).

To explore the functions of the isolated differential proteins, we analyzed protein sequences by mapping between InterPro domains and Gene Ontology (GO) terms, and the identified secreted proteins were categorized into five groups using InterPro entries annotated with GO terms: transporter activity (18%), protein binding (18%), nucleotide binding (36%), kinase activity (9%), and peptide activity (18%) (Figure 5a).

Notably, according to the KEGG database, several proteins were annotated to participate in cellular amino acid (25%) and carbohydrate metabolic (25%) processes, indicating that half of the differentially expressed proteins were associated with metabolic processes. These results suggested that metabolite metabolism plays an important role in the pathogenicity of *R. solanacearum*. The remaining proteins were grouped into various biological processes. Occasionally, a single protein was involved in several biological processes; thus, the total number of proteins (Figure 5b) was more than 24.

### 2.6. Abundance Patterns of the Differential Proteins in RsM and RsH in CPZ and Min Medium

**Figure 6:**
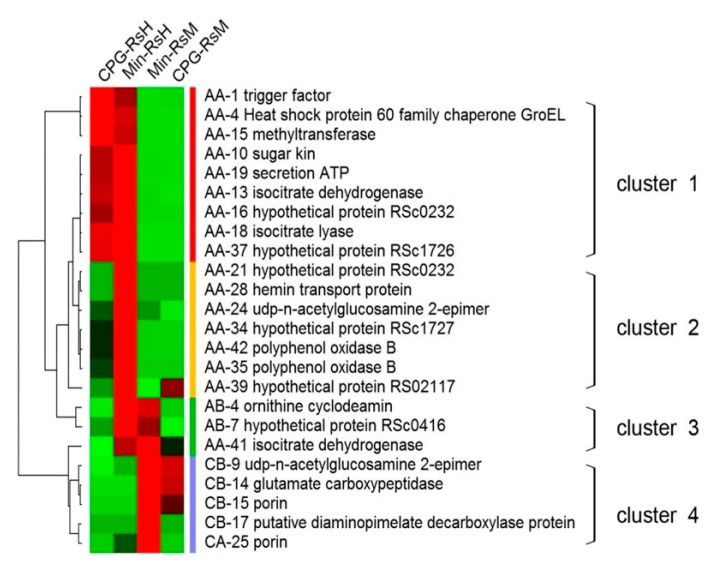
Hierarchical cluster analysis of the identified secreted proteins in different samples. The clustering analysis was performed with the PermutMatrix graphical interface after Z-score normalization of relative spot quantity peak values. Pearson′s distance and Ward′s algorithm were used for the analysis. Red and green represent increased and decreased protein abundance, respectively.

We also studied the abundance pattern of the 24 differential proteins. For this purpose, we clustered all the identified secreted proteins using Cluster 3.0 and TreeView. Notably, the results showed that the abundance of majority of the proteins (79.1%) were increased in RsH-Min, whereas in RsH-CPZ or -Min, most of the proteins abundance were decreased (66.7% and 83.4%, respectively) (Table S4, Figure 6). This result was not surprising, and we concluded that more pathways were activated in RsH-Min.

Of the abundance increased proteins in Min-RsH, three proteins (AB-4, AB-7, AA-41, or ornithine cyclodeamin, and hypothetical proteins RSc0416 and isocitrate dehydrogenase (IDL)), displayed abundances that also increased in RsM-Min (Figure 6). Moreover, four secreted protein spots showed the same abundance patterns in both RsM and RsH, namely CB9 (UDP-N-acetylglucosamine 2-epimerase, epsC), CB14 (glutamate carboxypeptidase), CB15 (porin), and CB17 (putative diaminopimelate decarboxylase protein). Some of the proteins, such as IDL and epsC, have been reported to be directly associated with bacterial pathogenicity. These proteins are considered to be strong potential factors that may directly or indirectly influence *R. solanacearum* virulence.

## 3. Discussion

In this study, we characterized two *R. solanacearum* strains, RsM (a non-pathogenic to Hawaii 7996, mildly aggressive strain) and RsH (a pathogenic, highly aggressive strain), which both infect the tomato cultivar “Hawaii 7996”. Phylogenetic analysis revealed that both strains belong to phylotype I, and are closely related. Furthermore, we conducted comprehensive proteomics analysis to identify proteins that are differentially expressed in RsM and RsH.

### 3.1. RsM and RsH Exhibited Different Virulence

*R. solanacearum* is defined as a species complex that reflects phenotypic and genotypic variation among species, which means “a cluster of closely related isolates whose individual members may represent more than one species” [22]. Traditionally *R. solanacearum* was divided into five races based on the host range, and into six biovars based on biochemical properties. A hierarchical classification system was developed by Fegan and Prior [22,23] based on *hrpB* and *endoglucanase (egl)* genes [22,23]. In this system, the *R. solanacearum* species complex was subdivided roughly into four phylotypes. Phylotype I includes biovars 3, 4, and 5, which primarily originate from Asia. Phylotype II includes biovars 1 and 2, largely from America. The strains of phylotype III mostly originate from Africa and the surrounding islands, and phylotype IV includes biovars 1 and 2, isolated primarily in Indonesia. Moreover, some strains identified in Australia and Japan also belonged to phylotype IV [24]. In this study, we performed multiplex PCR for the Internal transcribed spacer (ITS) region, and confirmed that both RsM and RsH belong to phylotype I, which is primarily found in Asia. Phylotypes can be subdivided into a range of sequevars, determined based on the egl-conserved sequence. The results showed >99% sequence similarity between these two strains, suggesting that RsM and RsH are closely related.

### 3.2. EPS as a Potential Factor Accounting for R. solanacearum Virulence

It has been accepted that two levels exist in plant immunity [25,26]. In the first stage, transmembrane pattern recognition receptors (PRRs) are employed that detect microbial- or pathogen-associated molecular patterns (MAMPS or PAMPs), and then via PRRs, PAMP-triggered immunity (PTI) is activated. The second stage is largely activated inside the cell; nucleotide-binding site leucine-rich repeat (NB-LRR) proteins, encoded by resistance (*R*) genes, are activated by pathogen effectors, thereby activating effector-regulated immunity (ETI) [3]. In the first stage, EPS, an indispensable virulence factor of *R. solanacearum*, physically blocks water flow in the xylem vessels, resulting in the development of the wilt symptom. Moreover, EPS also protects the bacterium from plant defense mechanisms. *R. solanacearum* additionally exerts its pathogenicity reply on the functionality of a Type 3 secretion system (T3SS) [27].

In the present study, UDP-N-acetylglucosamine 2-epimerase (epsC), a component of EPS I, which is considered to be the major factor accounting for the virulence of the pathogen, increases in abundance in both RsM (cluster 4) and RsH (cluster 2). Notably, the abundance of epsC protein spot was increased in RsH by Min medium, which mimics the limited nutritional environment of a plant, whereas this up-regulation was not observed in CPZ rich medium. Taking into account that epsC is an important bacterial protein involved in the EPS I biosynthesis pathway, which functions as a virulence factor, it is proposed that epsC was induced during the invasive process of *R. solanacearum* RsH. These results were in agreement with previous findings. McLoon [27] reported that a mutation in epsC leads to decreased EPS I production and, as a consequence, the bacterium strain exhibits an impaired biofilm formation phenotype, indicating that epsC plays an important role in EPS I production [27]. Additional evidence also revealed that site-directed mutants are unable to synthesize EPS, resulting in nearly avirulent *R. solanacearum* that fails to colonize plant xylem vessels [28,29,30,31].

*R. solanacearum* EPS has been demonstrated to play a different role in susceptibility to tomato host responses. *R. solanacearum* generally produces high amounts of EPS during bacterium-plant interactions, resulting in the wilt symptom in the infected plants. In our study, the abundance of epsC protein spot was increased in RsH by Min medium, and this finding was not surprising because EPS was abundantly produced at high densities inside tomato plant cells, which is critical for *R. solanacearum* wilt virulence. Nonetheless, we still do not specifically know whether EPS colonizes tomato stems by facilitated motility of *R. solanacearum* in plants, by blocking biofilm formation [30,32], or via activating/suppressing the ethylene (ET) and salicylic acid (SA) signaling pathways [33]. Thus, further studies are required to explore the detailed mechanism of pathogenicity induced by EPS.

### 3.3. Isocitrate Lyase (ICL) is More Abundant in RsH

Detecting the specific potential pathogenicity of RsH may help us to explore the factors associated with this lethal phenotype. In our study, the protein abundances of two isocitrate-related proteins, isocitrate dehydrogenase (IDH) and isocitrate lyase (ICL), were increased in RsH-Min. Interestingly, IDH and ICL compete for a common substrate, isocitrate. IDH and ICL convert isocitrate to 2-oxoglutatrate and glyoxylate, respectively. Moreover, IDH and ICL are crucial e nzymes during the TCA and glyoxylate cycles, particularly ICL, the accumulation of which is specifically limited to the glyoxylate cycle [34]. It is clear that the TCA cycle is the major energy-yielding metabolic pathway in the mitochondria, whereas the glyoxylate cycle is an important anaplerotic pathway of TCA, and is also involved in energy production and other biosynthetic processes. Overall, the TCA and glyoxylate cycles were enhanced in *R. solanacearum* strain RsH, indicating that more energy and intermediates, such as precursor nucleic acids and various amino acids, are required to confer the high virulence of *R. solanacearum* strains.

In addition, emerging evidence from diverse microbial pathogens has indicated that ICL plays an important role in conferring full bacterial virulence, and is essential for successful colonization in various plant species [33,34,35]. ICL participates in lipid utilization or gluconeogenesis [34]; thus, ICL-deficient mutants generally fail to, or only poorly utilize numerous intermediates, such as glycerol, lactate, and pyruvate, for their growth [36]. Wang [34] found that disruption of ICL1 in *Magnaporthe grisea* led to a reduction in appressorium formation and cuticle penetration, eventually leading to an overall mild degree of damage to rice leaves [34]. Idnurm and Howlett [37] reported that *ICL1*-insertion mutants of *Leptosphaeria maculans* exhibited significantly less virulence in canola *(Brassica napus)*, and that the reduced pathogenicity of the ICL1 mutant was because of its inability to utilize the carbon sources provided by the canola [37]. Similar results have been observed for multiple bacterial and fungal species, such as *Rhodococcus fascians*, *Candida albicans*, and *Mycobacterium tuberculosis* [35,36,37]. Bocsanczy [38] used two-dimensional difference gel electrophoresis (2D-DIGE) gel to compare the protein profiles of two isolates of *R. solanacearum* under high and low temperatures. Some proteins involved in different elements of metabolism, such as the glyoxylate cycle and leucine biosynthesis pathway, were identified [38].

In our study, the highly aggressive *R. solanacearum* strain, RsH, displayed induced ICL activity, suggesting that the glyoxylate cycle plays a potential positive role in *R. solanacearum* virulence. In the previous section, we discussed how EPS plays a potential role in high virulence via colonizing plant xylem vessels. Moreover, IDH and ICL also possibly play a crucial role in plant–bacteria interactions. Overall, we speculated that EPS and the glyoxylate cycle are two potential factors for successful colonization by R. solanacearum.

### 3.4. Membrane Proteins Possibly Play a Positive Role in Wilt Susceptibility in Tomatoes

In addition to the upregulated differential proteins in the highly aggressive strain RsH, we noticed that some proteins were significantly induced in the mildly aggressive strain RsM. For example, glutamate carboxypeptidase (GPC, spot CB14) and two porins (spot CB15 and CB25) abundance were increased in RsM. Interestingly, most of these proteins are membrane proteins.

Porins form hydrophilic channels, allowing the transport of molecules and required nutrients across the lipid bilayer membrane. In the mildly aggressive strain RsM, porin abundance was significantly increased, suggesting its role in pathogenicity. Some outer membrane porins, such as *Salmonella typhimurium* OmpD [39] and *Shigella flexneri* OmpC [40], have been reported to act as virulence factors. In the process of interaction between soil and plant roots, porins exert cytotoxic activity on cells that exhibit cell adhesion for pathogenicity [41]. However, the relationship between porins and pathogenicity is still unclear. GPC is another membrane protein that is also induced in the non-pathogenic isolate RsM. In mammalian cells, GPC is involved in various metabolic and signaling pathways via catabolizing small peptides [42,43]; however, the function of GPC in plants has been rarely studied. Thus, further validations with functional analyses are required to explore how GPC participates during the invasive process of *R. solanacearum*. Overall, our findings provide a new insight into how membrane proteins may be involved in plant–bacteria interactions.

## 4. Materials and Methods

### 4.1. Bacterial Strains and Growth Conditions

Two *R. solanacearum* strains used in this study, namely RsM and RsH, were isolated from wilted branches of tomatoes (*S. lycopersicum*) in Guangzhou (subtropical climate, south of China) in our laboratory, and it was found that RsH exhibited higher virulence than RsM. Bacterial strains were maintained and retrieved by plating on a TZC medium (casamino acids-pepton-glucose, CPG medium with 0.005% (*w*/*v*) 2,3,5-triphenyl tetrazolium chloride, TZC) [44]. Generally, strains on different plates were grown at 28 °C for two days. For the growth (propagation) rate test, a single colony was transferred to Min broth on a rotary shaker at 180 rpm. Cells in different growth time intervals (0 h, 8 h, 16 h, 24 h and 32 h) were harvested with sterile distilled water, and the optical density was measured.

### 4.2. RsM and RsH Infection

For the infiltration of the pathogens RsM and RsH, the bacterial suspensions were prepared in sterilized distilled water and adjusted to approximately 10^8^ colony-forming units (CFU) per mL (OD_600_ = 0.3). For the inactivated RsH infection, the RsH bacterial suspension was treated at 60 °C for 10 min. The tomato plants (cultivar ‘Hawaii 7996’) were grown in a greenhouse with a 16 h light/8 h dark cycle and 80% relative humidity at 25 °C. Four week-old tomato plants were inoculated with a bacterial suspension (10^7^ CFU/mL) onto the soil soak inoculation to check the response to RsM and RsH. Results were recorded after 24 h until 14 days after inoculation (dpi). Each assay was repeated in the successive trials.

### 4.3. Phylotype Identification

Firstly, total DNA was extracted from both strains using a Bacterial DNA kit (Suobaolai, Beijing, China) according to the manufacturer’s instructions. The phylotype affiliation of both strains was determined as described [22], by four forward primers (Nmult21:1F, Nmult21:2F, Nmult23: AF, Nmult22: InF), and a specific reverse primer for each phylotype by multiplex PCR. Furthermore, the *endoglucanase (egl)* and *transcriptional regulator (h pB)* genes were amplified by the primer pairs Endo-F/Endo-R and RSh pBF/RSh pBR, respectively. The sequences of primers are listed in Supplementary Tables S1 and S2. The products of *egl* and *h pB* were sequenced by Sangon Bitotech Co.; Ltd. (Guangzhou, China). Each 20 μL PCR reaction contained 10.2 μL water, 2.0 μL 10 × buffer (Mg^2+^), 2 μL dNTPs (10 mM), 2 μL each of upstream and downstream primers (1 μM), and 0.5 μL *Taq* DNA polymerase (10 U/μL). The PCR amplification was performed with the following program: 94 °C for 5 min followed by 36 cycles of 94 °C for 30 s, 55 °C for 30 s, and 72 °C for 1 min, with a final extension at 72 °C for 10 min. The PCR products were sent to Sangon Bitotech Co.; Ltd. (Guangzhou, China) for sequencing. Phylogenetic analysis was applied using MEGA5 (Version4.0, odesign Institute, Arizona State University, USA, https://www.megasoftware.net) via the neighbor-joining method [45].

### 4.4. Protein Extraction from RsM and RsH

Firstly, the bacterial strains were grown on TZC medium on 28 °C for two days, and subsequently a signal colony was transferred to CPZ (Peptone,10 g/L, dextrose,10 g/L, casamino acid, 10 g/L, 1% TTZ, 5 mL/L) and Min [46] broth on a rotary shaker at 180 rpm overnight, until OD_600_ = 0.6. One gram of trichloracetic acid (TCA) was added to 10 mL of cells at a final concentration of 10% (*w*/*v*), then the mixture was stored at −20 °C for 2 h, before being collected by centrifugation at 18,000× rpm/min for 10 min at 4 °C. The rest of the procedure was carried out as described earlier [47,48]. Finally, the supernatant was subjected to electrophoresis. The protein concentration of each sample was determined by Bradford assay using BSA as a control.

4.5. 2D Gel Electrophoresis

A total of 400 μg of each protein sample was dissolved in 210 μL of rehydration solution containing 7 M urea, 2 M thiourea, 4% (*w*/*v*) 3-((3-cholamidopropyl)dimethylammonium)-1-propanesulfonate (CHAPS), 0.5% (*v*/*v*) immobilized pH gradient (IPG) buffer (GE Healthcare, Uppsala, Sweden), 40 mM Dithiothreitol (DTT), and 0.002% (*w*/*v*) Bromophenol Blue. The proteins were separated by isoelectric focusing (IEF) using the GE Ettan IPGphor 3 (GE Healthcare) with a total of 75,000 Vh, as follows: a low initial voltage of 300 V, stepwise increases to 1000 V for 1 h, and stepwise increases to 8000 V up to 75,000 Vh.

Before running the second gel, the gel strips were equilibrated for 15 min in equilibration buffer A, containing 0.1 M Tris-HCl, 2% (*w*/*v*) SDS, 6 M urea, 30% (*w*/*v*) glycerol, and 0.1 M DTT at pH 8.8, and then for another 15 min in equilibration buffer B in which the 0.1 M DTT was replaced by 0.25 M iodoacetamide. Sodium dodecyl sulfate polyacrylamide gel electrophoresis (SDS-PAGE) was performed using a BioRad Vertical Electrophoresis System (Bio-Rad, Hercules, CA, USA). After equilibration, the IPG strips were applied to the surface of the second-dimension vertical 12.5% SDS-PAGE gels, which could separate proteins in the 10–100 kDa range. The gels were stained in 0.066% Coomassie brilliant blue (CBB) G-250 overnight and destained in H_2_O.

### 4.6. Gel Imaging and Data Analysis

The gel images were analyzed with PDQuest software (Version 8.0, Bio-Rad, Hercules, CA, USA) for spot detection, gel matching, and statistical analysis of spots. To compare spots, the sum of the spot densities on each gel was normalized. The software calculated individual spot “volumes” in each gel by density/area integration. After detection of spots by PDQuest software, the files were also inspected manually to assess the accuracy of the computer-generated images. The selection of differentially expressed protein spots for MS-MS analysis was based on fold-changes of >2.0 in abundance. Only spots present in all the replicate gels were considered for subsequent analysis.

### 4.7. MALDI-TOF/MS-MS Identification of Differential Protein Spots

Spots showing statistically significant changes were cut out manually from the gels and washed twice with ultrapure water in sterilized Eppendorf tubes. Then, the protein spots were washed with 50% acetonitrile (ACN) in 25 mM NH_4_HCO_3_. The gels were dehydrated twice with 50% ACN followed by 100% ACN, and finally dried in a vacuum centrifuge. The proteins were digested overnight with trypsin (Promega, Madison, WI, USA, 0.02 μg/μL in 25 mM NH_4_HCO_3_) at 37 °C. The supernatant of the resulting peptides was washed with 0.1% trifluoroacetic acid (TFA) in 67% ACN. The extracts were lyophilized and collected by centrifugation for MS analysis.

All of the dried samples were analyzed using a 4800 MALDI-TOF/MS-MS Proteomics Analyzer (Applied Biosystems, Lincoln Centre Drive Foster City, CA, USA). The positive ion and automatic data acquisition modes were used for data collection, and TOF spectra were collected over the mass range of 800–3500 Da. A maximum of 10 precursors per spot with a minimum signal/noise ratio of 50 were selected for data-dependent MS/MS analysis.

### 4.8. Differential Proteins Analysis

The primary and secondary mass spectrum data were analyzed with the GPS Explorer software (Version 3.6, Applied Biosystems, Lincoln Centre Drive Foster City, CA, USA). Database searches were performed with the MASCOT software (Version 2.1.03, Matrix Science, London, UK) using the non-redundant protein sequence database NCBInr Taxonomy, Bacteria (Eubacteria, https://www.ncbi.nlm.nih.gov/genome/microbes/) to identify proteins. The search parameters were set as follows: taxonomy, Viridiplantae (Green Plants; released June 2013; 1437595 sequences, https://www.ncbi.nlm.nih.gov/taxonomy), trypsin was used as the digestive enzyme, up to one missed cleavage was allowed, carbamidomethylation (Cys) and oxidation (Met) were variable modifications and there were no fixed modifications, peptide tolerance was 50 ppm, and MS/MS tolerance was 0.06 Da. Only significant hits, as defined by the MASCOT probability analysis (*p* < 0.05), were accepted [49].

Homology searches (BlastP) of unique sequences and functional annotation by gene ontology terms (GO), InterPro terms (InterProScan, EBI), enzyme classification codes (EC), and metabolic pathways (KEGG, Kyoto Encyclopedia of Genes and Genomes) were performed using the BLAST2GO software suite v2.6.6 (Valencia, Spain).

## Figures and Tables

**Table 1:** List of the identified differentially expressed proteins in four samples.

Spot Number	Accession	Protein Name	Score	Theoretical Mr(kDa)/p*I*	Observed Mr(kDa)/p*I*	Organism	Protein Sequence Coverage
**cluster 1**							
AA-1	17546429	trigger factor	419	50.093/5.17	51.79/5.05	*R. solanacearum* GMI1000	17%
AA-4	469776741	Heat shock protein 60 family chaperone GroEL	172	57.231/5.21	56.79/5.14	*R. solanacearum* FQY_4	6%
AA-10	17549499	sugar kinase	503	32.734/5.57	32.67/5.77	*R. solanacearum* GMI1000	30%
AA-13	17547209	isocitrate dehydrogenase	376	45.827/5.65	45.45/5.98	*R. solanacearum* GMI1000	14%
AA-15	17548395	methyltransferase	412	31.336/5.53	29.34/6.05	*R. solanacearum* GMI1000	23%
AA-16	17544951	hypothetical protein RSc0232	352	30.243/6.1	30.3/6.1	*R. solanacearum* GMI1000	22%
AA-18	17546077	isocitrate lyase	115	49.094/6.06	49.87/6.2	*R. solanacearum* GMI1000	6%
AA-19	17549306	secretion ATP	499	49.500/5.91	49.67/6.32	*R. solanacearum* GMI1000	18%
AA-37	17546445	hypothetical protein RSc1726	631	22.449/6.29	22.07/7.01	*R. solanacearum* GMI1000	56%
**cluster 2**							
AA-21	17544951	hypothetical protein RSc0232	848	30.243/6.1	29.75/6.27	*R. solanacearum* GMI1000	43%
AA-24	17549238	udp-n-acetylglucosamine 2-epimerase	599	41.656/5.95	38.3/6.38	*R. solanacearum* GMI1000	25%
AA-28	17548464	hemin transport protein	362	41.267/6.23	40.7/6.48	*R. solanacearum* GMI1000	16%
AA-34	17546446	hypothetical protein RSc1727	350	20.751/6.15	20.46/6.64	*R. solanacearum* GMI1000	29%
							
AA-35	17545056	polyphenol oxidase B	454	54.474/7.31	50.52/7.04	*R. solanacearum* GMI1000	14%
AA-39	17549780	hypothetical protein RS02117	996	16.136/6.74	17.48/6.95	*R. solanacearum* GMI1000	66%
AA-42	17545056	polyphenol oxidase B	152	54.474/7.31	50.52/7.3	*R. solanacearum* GMI1000	7%
**cluster 3**							
AA-41	17547209	isocitrate dehydrogenase	231	45.827/5.65	46.39/5.6	*R. solanacearum* GMI1000	11%
AB-4	17548639	ornithine cyclodeamin	896	37.988/5.53	37.65/5.66	*R. solanacearum* GMI1000	36%
AB-7	17545135	hypothetical protein RSc0416	168	20.629/5.48	24.81/5.72	*R. solanacearum* GMI1000	12%
**cluster 4**							
CB-9	17549238	udp-n-acetylglucosamine 2-epimerase	599	41.656/5.95	38.4/6.28	*R. solanacearum* GMI1000	27%
CB-14	17548494	glutamate carboxypeptidase	202	41.526/6.42	40.47/6.62	*R. solanacearum* GMI1000	13%
CB-15	17547574	porin	305	41.269/8.46	38/6.6	*R. solanacearum* GMI1000	10%
CB-17	4.7E+08	putative diaminopimelate decarboxylase protein	265	45.900/6.47	46.98/6.98	*R. solanacearum* FQY_4	13%
CA-25	17545414	porin	138	51.791/8.54	49/7.2	*R. solanacearum* GMI1000	8%

## Supplementary Materials

Supplementary materials can be found at http://www.mdpi.com/1422-0067/19/8/2444/s1.
