# Supplementary material for: Comparative Proteomic Analysis of Two Ralstonia solanacearum Isolates Differing in Aggressiveness
Source: Int J Mol Sci. 2018 Aug 18;19(8):2444. doi: 10.3390/ijms19082444 (PMC6121549; doi:10.3390/ijms19082444)
Supplement: Supplementary file 1 [file ijms-19-02444-s001.zip › Suppmental figures.pdf]

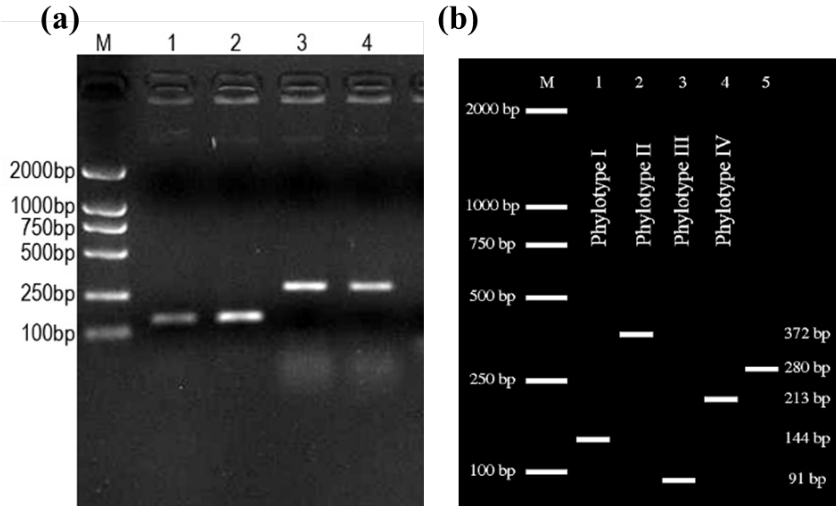

FigureS1 (a)Electrophoretic analysis of polymerase chain reaction(PCR)-amplified DNA from R. solanacearum strains RsM and RsH using primer pairs(Nmult21F/Nmult21RR and 759/760)(Fegan and Prior, 2005). LanesM. DNA Marker DL2000; line 1.a representive phylotype I in R. solanacearum strain RsM ; line2. a representive phylotype I in R. solanacearum strain RsH I; line3. R. solanacearum-specific band in RsM; line4. R. solanacearum-specific band in RsH, the arrow represent the Phylotype I specific band (166bp); (b) PCR products of different phylotypes.

NW Score	Identities	Gaps	Str
1332	666/666(100%)	0/666(0%)	Plu:

```
Query  1    ACCGACACCACGACCCTGAAGACGGCCGCCACCACCTCGATCTCGCCGTTGTGGCTCACC  60
            ||||||||||||||||||||||||||||||||||||||||||||||||||||||||||||
Sbjct  1    ACCGACACCACGACCCTGAAGACGGCCGCCACCACCTCGATCTCGCCGTTGTGGCTCACC  60

Query  61   GTCGCCAAGGACAGCGCGGCGTTCACGGTGAGCGGCACGCGCACGGTGCGCTATGGCGCC  120
            ||||||||||||||||||||||||||||||||||||||||||||||||||||||||||||
Sbjct  61   GTCGCCAAGGACAGCGCGGCGTTCACGGTGAGCGGCACGCGCACGGTGCGCTATGGCGCC  120

Query  121  GGCAGCGCGTGGGTGGCGAAGAGCATGTCCGGCACAGGCCAGTGCACCGCCGCCTTCTTC  180
            ||||||||||||||||||||||||||||||||||||||||||||||||||||||||||||
Sbjct  121  GGCAGCGCGTGGGTGGCGAAGAGCATGTCCGGCACAGGCCAGTGCACCGCCGCCTTCTTC  180

Query  181  GGCAAGGATCCGGCGGCCGGTGTCGCCAAGGTGTGCCAAGTGGCGCAGGGCACGGGCACC  240
            ||||||||||||||||||||||||||||||||||||||||||||||||||||||||||||
Sbjct  181  GGCAAGGATCCGGCGGCCGGTGTCGCCAAGGTGTGCCAAGTGGCGCAGGGCACGGGCACC  240

Query  241  CTGCTGTGGCGCGGCGTCAGCCTGGCCGGCGCCGAGTTCGGGGAGGGCAGCCTGCCGGGC  300
            ||||||||||||||||||||||||||||||||||||||||||||||||||||||||||||
Sbjct  241  CTGCTGTGGCGCGGCGTCAGCCTGGCCGGCGCCGAGTTCGGGGAGGGCAGCCTGCCGGGC  300

Query  301  ACCTACGGGAGCAACTACATCTATCCGTCCGCCGACAGCGCGACCTACTACAAGAACAAG  360
            ||||||||||||||||||||||||||||||||||||||||||||||||||||||||||||
Sbjct  301  ACCTACGGGAGCAACTACATCTATCCGTCCGCCGACAGCGCGACCTACTACAAGAACAAG  360

Query  361  GGCATGAACCTGGTGCGCCTGCCGTTCCGCTGGGAGCGGCTGCAGCCCACGCTCAACCAG  420
            ||||||||||||||||||||||||||||||||||||||||||||||||||||||||||||
Sbjct  361  GGCATGAACCTGGTGCGCCTGCCGTTCCGCTGGGAGCGGCTGCAGCCCACGCTCAACCAG  420

Query  421  GCGCTCGACGCGAACGAGCTGTCGCGCCTGACCGGGTTCGTCAACGCCGTGACGGCGGCC  480
            ||||||||||||||||||||||||||||||||||||||||||||||||||||||||||||
Sbjct  421  GCGCTCGACGCGAACGAGCTGTCGCGCCTGACCGGGTTCGTCAACGCCGTGACGGCGGCC  480

Query  481  GGCCAGACGGTGCTGCTCGATCCGCACAACTACGCGCGCTACTACGGCAACGTGATCGGC  540
            ||||||||||||||||||||||||||||||||||||||||||||||||||||||||||||
Sbjct  481  GGCCAGACGGTGCTGCTCGATCCGCACAACTACGCGCGCTACTACGGCAACGTGATCGGC  540

Query  541  TCGAGCGCGGTGCCCAACAGCGCGTACGCCGATTTCTGGCGGCGCGTGGCCACCCAGTTC  600
            ||||||||||||||||||||||||||||||||||||||||||||||||||||||||||||
Sbjct  541  TCGAGCGCGGTGCCCAACAGCGCGTACGCCGATTTCTGGCGGCGCGTGGCCACCCAGTTC  600

Query  601  AAGGGCAATGCCCGCGTCATCTTCGGGCTGATGAACGAGCCCAATTCGATGCCGACCGAG  660
            ||||||||||||||||||||||||||||||||||||||||||||||||||||||||||||
Sbjct  601  AAGGGCAATGCCCGCGTCATCTTCGGGCTGATGAACGAGCCCAATTCGATGCCGACCGAG  660

Query  661  CAGTGG  666
            ||||||
Sbjct  661  CAGTGG  666
```

Figure S2 Comparison of *egl* gene between and *R. solanacearum* strains RsM(upper sequence) and

RsH(lower sequence).

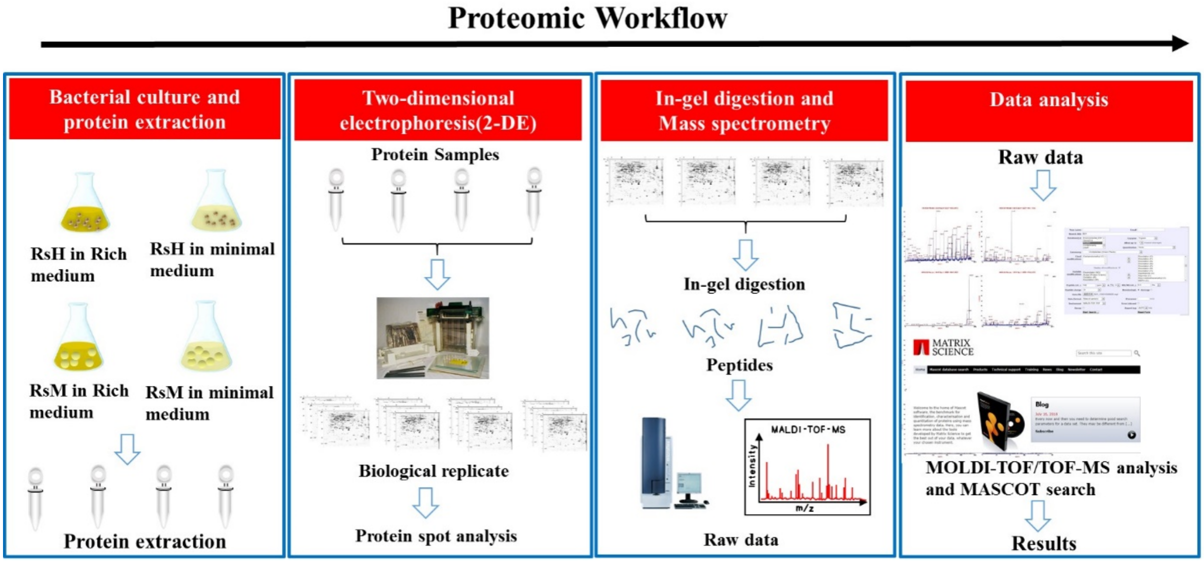

Figure S3 Experimental system of proteomic workflow.

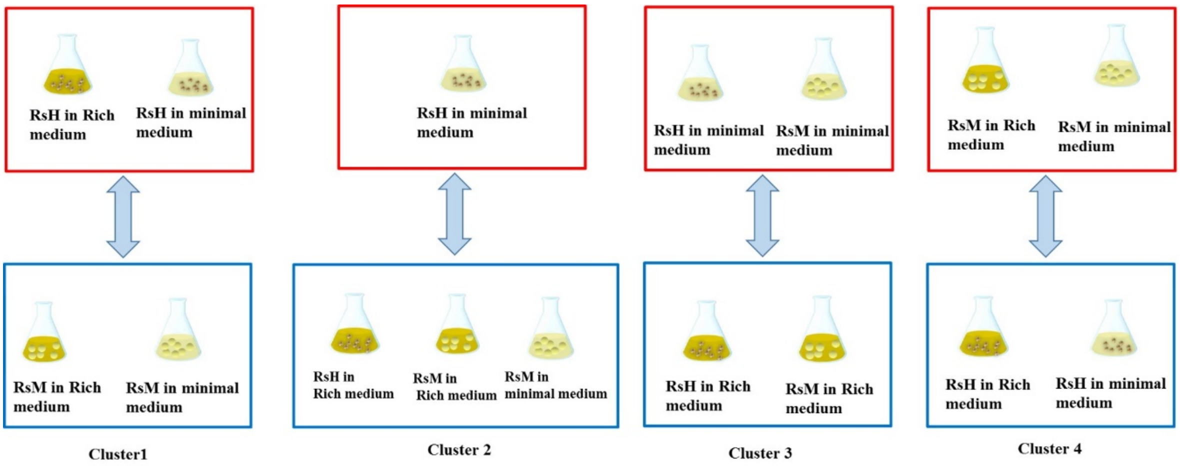

Figure S4 a brief layout of the comparison based on the protein abundance in different samples. Red box represent the protein abundance increased compared with those in blue box samples.
