# Supplementary material for: Comparative Proteomic Analysis of Two Ralstonia solanacearum Isolates Differing in Aggressiveness
Source: Int J Mol Sci. 2018 Aug 18;19(8):2444. doi: 10.3390/ijms19082444 (PMC6121549; doi:10.3390/ijms19082444)
Supplement: Supplementary file 1 [file ijms-19-02444-s001.zip › Suppmental tables.pdf]

Table S1 Primer used for phylotype specific multiplex PCR

primer	Sequence(5'→3')	Specificity	length
Nmult21:1F	CGTTGATGAGGCGCGCAATTT	Phylotype I	144bp
Nmult21:2F	AAGTTATGGACGGTGGAAGTC	Phylotype II	372bp
Nmult23:AF	ATTACSAGAGCAATCGAAAGATT	Phylotype III	91bp
Nmult22:InF	ATTGCCAAGACGAGAGAAGTA	Phylotype IV	213bp
Nmult22:RR	TCGCTTGACCCTATAACGAGTA	All Phylotypes	
759	GTCGCCGTCAACTCACTTTCC	Specific Phylotype	280bp
760	GTCGCCGTCAGCAATGCGGAATCG		

Table S2 Primer sequences of *egl* and *hrpB* genes

gene	primer	length	Locus name
egl	Endo-F: 5'- ATGCATGCCGCTGGTCGCCGC -3'	840 bp	RSp0162
	Endo-R: 5'- GCGTTGCCCGGCACGAACACC -3'		
hrpB	RShrpBF: 5' TGCCATGCTGGGAAACATCT -3'	1400 bp	RSp0873
	RShrpBR: 5'- GGGGGCTTCGTTGAACTGC -3'		
